# The Transcriptional Control of Iron Homeostasis in Plants: A Tale of bHLH Transcription Factors?

**DOI:** 10.3389/fpls.2019.00006

**Published:** 2019-01-18

**Authors:** Fei Gao, Kevin Robe, Frederic Gaymard, Esther Izquierdo, Christian Dubos

**Affiliations:** BPMP, CNRS, INRA, Montpellier SupAgro, University of Montpellier, Montpellier, France

**Keywords:** basic helix loop helix, bHLH, iron, homeostasis, *Arabidopsis thaliana*

## Abstract

Iron is one of the most important micronutrients in plants as it is involved in many cellular functions (e.g., photosynthesis and respiration). Any defect in iron availability will affect plant growth and development as well as crop yield and plant product quality. Thus, iron homeostasis must be tightly controlled in order to ensure optimal absorption of this mineral element. Understanding mechanisms governing iron homeostasis in plants has been the focus of several studies during the past 10 years. These studies have greatly improved our understanding of the mechanisms involved, revealing a sophisticated iron-dependent transcriptional regulatory network. Strikingly, these studies have also highlighted that this regulatory web relies on the activity of numerous transcriptional regulators that belong to the same group of transcription factors (TF), the bHLH (basic helix-loop-helix) family. This is best exemplified in Arabidopsis where, to date, 16 bHLH TF have been characterized as involved in this process and acting in a complex regulatory cascade. Interestingly, among these bHLH TF some form specific clades, indicating that peculiar function dedicated to the maintenance of iron homeostasis, have emerged during the course of the evolution of the green lineage. Within this mini review, we present new insights on the control of iron homeostasis and the involvement of bHLH TF in this metabolic process.

## Introduction

Iron (Fe) is one of the most important micronutrient elements in plants as it is involved in many cellular functions (e.g., photosynthesis and respiration). Any defect in Fe availability will impact plant growth and development as well as crop yield and plant product quality (Briat et al., 2015).

In order to cope with Fe shortage and recover Fe from soil, where it is in poorly available forms, plants have evolved two strategies. The first one, strategy I, is used by all dicots and non-graminaceous monocots. This strategy consists in rhizosphere acidification via proton extrusion in order to promote Fe solubility and involves proton-ATPase such as AHA2. The secretion by the root of Fe-mobilizing phenolic compounds facilitates this process (Fourcroy et al., 2014, 2016). Fe^3+^ is thus reduced into Fe^2+^ by ferric chelate reductases, such as FRO2 (FERRIC REDUCTION OXIDASE 2), prior to being transported across the rhizodermis cell membranes by IRT1 (IRON-REGULATED TRANSPORTER 1) (Brumbarova et al., 2015). The second strategy, strategy II, is used by graminaceous species. This strategy consists in releasing phytosiderophores into the rhizosphere to chelate Fe^3+^ (Nozoye et al., 2011). Fe^3+^-phytosiderophores chelates are then transported into the roots by the YELLOW STRIPE 1 transporter (Curie et al., 2001). If the machinery allowing plant Fe uptake from the soil is central for the maintenance of Fe homeostasis, this is indeed not the sole mechanism involved in this process. It also necessitates several genes encoding proteins involved in Fe transport, compartmentation and storage, at the cellular and subcellular levels, throughout the entire plant body. Such complex mechanism must thus be tightly regulated in order to avoid any physiological situation that would be deleterious to the plant.

**FIGURE 1:**
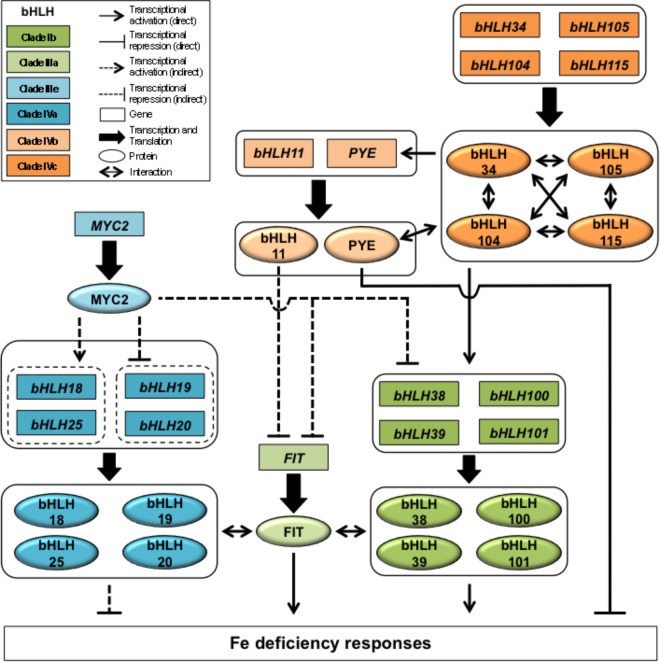
bHLH-dependent transcriptional regulatory network controlling the iron deficiency response in *Arabidopsis thaliana*.

How, at the molecular level, plants control Fe homeostasis has thus been a critical question for several years. This question has been mostly addressed by studying plant response to Fe deficiency, in particular in the model plant *Arabidopsis thaliana*. These studies have highlighted that such response involves an intricate network of basic helix-loop-helix (bHLH) transcription factors (TF) (Figure 1). bHLH proteins form one of the largest family of TFs found in plants that act as homo- or heterodimers to regulate the expression of their target genes. In Arabidopsis, 133 members have been identified and divided into 12 clades (Heim et al., 2003). From what is known on the role played by several members of this family of TFs in plants, it appears that their participation in the control of Fe homeostasis is unique by the number of individual TFs and different clades that are involved as well as by the intricacy of the network they form.

## The Molecular Regulation of Plant Iron Homeostasis

### The bHLH Regulatory Network

Upstream from the regulatory network involved in Arabidopsis Fe deficiency response are four bHLH TFs belonging to the clade IVc, namely bHLH34, bHLH104, bHLH105/ILR3 (IAA-LEUCINE RESISTANT3), and bHLH115 (Figure 1). These four TFs, shown to interact *in vivo* in the form of homo- or heterodimers, act as transcriptional activators of the plant response to Fe deficiency and display partial redundant activities (Zhang et al., 2015; Li et al., 2016; Liang et al., 2017). Clade IVc bHLH targets consist of *bHLH47/PYE* (*POPEYE*; clade IVb) and four clade Ib bHLH genes (*bHLH38*, *bHLH39*, *bHLH100*, and *bHLH101*) (Colangelo and Guerinot, 2004; Wang et al., 2007; Yuan et al., 2008; Wang N. et al., 2013; Zhang et al., 2015). PYE acts as a transcriptional repressor. For example, PYE was shown to inhibit the expression of *NAS4* (*NICOTIANAMINE SYNTHASE 4*), a key gene involved in phloem-based transport of Fe to sink organs, and *FRO3*, a Fe reductase located in root vasculature mitochondria (Jeong and Connolly, 2009; Klatte et al., 2009; Long et al., 2010). Interestingly, PYE can interact *in vivo* with bHLH104, ILR3, and bHLH115 (Long et al., 2010; Zhang et al., 2015). Whether or not these interactions play a role in the plant response to Fe deficiency or in the control of Fe homeostasis is an important question that remains to be elucidated. In contrast, bHLH38, bHLH39, bHLH100, and bHLH101 are partially redundant proteins that function at the root epidermis as positive regulators of *FRO2* and *IRT1*. This activity relies on their interaction with FIT (Fe-deficiency induced transcription factor), a clade IIIa bHLH (Colangelo and Guerinot, 2004; Yuan et al., 2008; Wang N. et al., 2013; Maurer et al., 2014). *FIT* expression is likely controlled, at least in part, by a feed forward regulatory loop involving bHLH39 (Naranjo-Arcos et al., 2017). bHLH6/MYC2 (clade IIIe) is a master regulator of the jasmonic acid (JA) signaling pathway whose activity differentially affects the expression clade IVa bHLH (bHLH18, bHLH19, bHLH20, and bHLH25) genes to modulate FIT protein accumulation (Cui et al., 2018). This mechanism relies on the direct interaction of clade IVa bHLHs with FIT in order to promote its degradation via the 26S proteasome pathway (Cui et al., 2018). In addition, MYC2 is a JA-dependent repressor of *FIT* and clade Ib bHLH genes expression, hence inhibiting FIT-dependent Fe uptake machinery at the transcriptional and posttranslational levels (Cui et al., 2018). Interestingly, *bHLH11*, another clade IVb bHLH, was recently proposed to be a negative regulator of FIT-dependent Fe uptake mechanism effecting Fe levels in Arabidopsis plants (Tanabe et al., 2018).

Altogether, this is 16 bHLH TFs out of the 133 present in Arabidopsis that have been identified as involved in the control of the Fe deficiency response, which represent more than 12% of the members of this large family of TFs (Heim et al., 2003). Indeed, orthologous members from the above-described clades were identified in other strategy I plant species such as tomato, apple, or soybean (Ling et al., 2002; Du et al., 2015; Zhao et al., 2016b; Li et al., 2018). In soybean, it is likely that the orthologs of *FIT* (*GmbHLH57*) and clade Ib bHLH (*GmbHLH300*) genes may also play a role in nodules, a tissue where several enzymes involved in symbiotic nitrogen fixation require Fe for their activity (Tang et al., 1990; O’Hara, 2001; Li et al., 2018). With the exception of FIT, which is specific to strategy I plants, orthologs of several bHLH are present in strategy II plants, indicating that the regulatory cascade controlling plant response to Fe deficiency is mostly conserved within the plant kingdom. For instance, orthologs of clade Ib (*OsIRO2*), IVc (*OsPRI1*), *PYE* (*OsIRO3*) and *MYC2* (*OsMYC2*) genes have been characterized in rice (Ogo et al., 2007; Zheng et al., 2010; Ogawa et al., 2017; Zhang et al., 2017). However, no orthologous bHLH TF in strategy I plants has been described so far for OsbHLH133, another regulator of the Fe deficiency response in rice (Wang L. et al., 2013). Is OsbHLH133 function specific to strategy II plants as it is the case for FIT in strategy I plants? Protein sequence analysis tends to indicate that OsbHLH133 is closely related to the bHLH clade VIIIc and thus it might be that this clade plays a role in the control of Fe homeostasis in both strategy I and II plants. If this hypothesis is verified, it will certainly render more complicated our understanding of this transcriptional regulatory network. Indeed, it is not the complexity of this network that is intriguing considering that any defect in the control of Fe homeostasis might be deleterious to the plants. The main question is why so many bHLHs are involved in this process? If it is difficult to answer this question, the involvement of a large number of TFs from one family in a specific process has already been described. This is the case with the R2R3-MYB family where at least 19 members out of 122 (about 16%) are involved in the control of the phenylpropanoid pathway (Dubos et al., 2010; Zamioudis et al., 2014; Xu et al., 2015). From these observations it would be tempting to speculate that during the course of the evolution of the green lineage, TF families have evolved specialized roles, in which plant Fe homeostasis would be mostly regulated by TFs belonging to the bHLH gene family.

How these bHLH TFs interact with the *cis*-regulatory sequences usually present in the promoter of their target genes is an important question considering that (i) each bHLH must specifically recognized its own target and (ii) the number of bHLH involved in this complex network. Indeed, it is well known that bHLH TF bind to specific DNA motifs (*CANNTG*) named *E-box*, and in particular to the canonical *CACGTG* sequence, named *G-box* (De Masi et al., 2011). For instance, chromatin immunoprecipitation assays showed that PYE, bHLH104, bHLH115, ILR3, and FIT preferentially bound to the promoter of their target genes in region that contain *E-box* or *G-box* (Long et al., 2010; Zhang et al., 2014, 2015; Liang et al., 2017). Similar observations were made, using biochemical approaches, for MdbHLH104 and OsPRI1 (Zhao et al., 2016a; Zhang et al., 2017). Interestingly, it was demonstrated that the genomic regions flanking *E-box* binding sites influence the DNA binding specificity of TFs (Gordan et al., 2013; Ezer et al., 2017). This is for example the case for OsIRO2 that binds preferentially to *CACGTGG* motif (Ogo et al., 2007). Hence, despite the fact that several direct target genes have been identified for most of the bHLH involved in the transcriptional control of Fe homeostasis and the fact that it is possible to infer the *E-box* sequence recognized by a given bHLH dimer based on bHLH domain compositions (De Masi et al., 2011), very little is known on the actual bHLH/DNA interactions.

### The Other Actors Involved in the Transcriptional Control of Plant Fe Homeostasis

Additional TFs, from several gene families, involved in the control of Fe homeostasis in both strategy I and strategy II plant species, have also been characterized.

MYB10 and MYB72, two R2R3-MYB TFs whose expression is partially dependent on FIT, are involved in Fe acquisition and distribution by notably regulating the expression of *BGLU42* and *NAS4* (Palmer et al., 2013; Stringlis et al., 2018). A closely related apple R2R3-MYB, MdMYB58, was recently reported as potentially involved in the control of Fe transport and tissue partitioning. It is proposed that MdMYB58 activity is repressed by its heterodimerization with MdSAT1, a clade IVa bHLH (Wang et al., 2018). WRKY46 plays a critical role in Fe translocation from root to shoot by directly repressing the expression of *VITL1/VTL1* (*vacuolar iron transporter like 1*) (Yan et al., 2016). ERF4 and ERF72 (AP2/ERF TFs) play negative roles in plant response to Fe deficiency by inhibiting the expression of genes involved in Fe uptake such as *IRT1* or *AHA2* (Liu et al., 2017a,b). Two TFs (EIL family) involved in ethylene signaling (EIN3, ETHYLENE INSENSITIVE 3 and EIL1, ETHYLENE INSENSITIVE 3 LIKE 1) and ZAT12 (a C2H2-type plant-specific zinc finger TF) are also involved by modulating FIT stability (Lingam et al., 2011; Le et al., 2016). Two MYB-CC TFs (PHR1, PHOSPHATE STARVATION RESPONSE 1 and PHL1, PHR1-LIKE 1), that play a central role in the phosphate deficiency response, regulate the expression of the main Fe storage ferritin gene in Arabidopsis (*AtFER1*), indicating that they act as integrators of both the phosphate and Fe signaling pathways (Bournier et al., 2013).

IDEF1 (ABI3/VP1 family) is an early regulator of Fe deficiency response in rice that directly binds to divalent metals suggesting that IDEF1 is a cellular sensor of metal ion balance caused by changes in Fe availability (Kobayashi et al., 2009, 2012). IDEF2 (NAC family) and OsARF16 (ARF family) play also critical roles in the control of Fe homeostasis in rice by modulating the expression of Fe-related genes and by integrating auxin signals, respectively (Ogo et al., 2008; Shen et al., 2015).

## The Molecular Regulation of the Transcriptional Regulatory Cascade Controlling Iron Homeostasis

### Post-translational Regulation of bHLH TFs

Iron deficiency results in a transcriptional response that leads to the activation of the Fe uptake machinery, which could lead to a Fe overload if it becomes suddenly available or to a toxic overaccumulation of other divalent metals (e.g., Zn, Mn, and Cd) due to the low specificity of IRT1. To cope with this, plants have developed posttranslational mechanisms such as the continuous recycling of IRT1 (Barberon et al., 2011, 2014). In addition, in Arabidopsis, IRT1 phosphorylation and ubiquitination leads to its internalization and subsequent degradation, a process that is triggered by direct binding of IRT1 to non-Fe metals (Ivanov et al., 2014; Dubeaux et al., 2018). However, maintaining Fe homeostasis requires also to tightly regulating, at the posttranslational level, the TFs involved in this process.

Fe-deficiency induced TF posttranslational regulation has been extensively investigated in Arabidopsis. For instance, it was shown that FIT heterodimerization with clade IVa bHLH promotes its degradation via the 26S proteasome pathway, whereas its interaction with clade Ib bHLH promotes its stability (Cui et al., 2018). FIT interaction with EIN3 or EIL1 also promotes its stability and contributes to Fe acquisition during the early stage of Fe deficiency (Lingam et al., 2011). In contrary, FIT interaction with ZAT12 has an inhibitory effect on its function when plants are grown under Fe sufficiency or prolonged Fe deficiency conditions (Le et al., 2016).

Several ubiquitin E3 ligases targeting bHLH TFs involved in the response to Fe deficiency have been characterized. *BRUTUS* (*BTS*), whose expression is induced upon Fe deficiency, is thought to be a Fe-sensing negative regulator in Arabidopsis (Selote et al., 2015). *In vitro* analyses suggest that BTS restricts the accumulation of ILR3 and bHLH115 through its RING E3 ligase activity and mediates their 26S proteasomal degradation (Selote et al., 2015). Whether the interaction of bHLH104 with BTS participate to its degradation remains to be demonstrated (Selote et al., 2015). The identification of two BTS ortologues in rice, namely OsHRZ1 [HEMERYTHRIN MOTIF-CONTAINING REALLY INTERESTING NEW GENE (RING) AND ZINC-FINGER PROTEIN 1] and OsHRZ2, suggests that BTS function is conserved in strategy II plants (Kobayashi et al., 2013). In addition to BTS, two closely related RING E3 ligases named BTSL1 and BTSL2 (BRUTUS LIKE 1 and 2) are proposed to negatively regulate the Fe deficiency responses by directly targeting FIT, leading to its degradation (Sivitz et al., 2011; Hindt et al., 2017; Rodriguez-Celma et al., 2018). Interestingly, it was shown in apple that a cullin-based E3 ligase mechanism, involving two BTB-TAZ proteins (MdBT1 and MdBT2) and MdCUL3, target MdbHLH104 for ubiquitin-dependent degradation via the 26S proteasome (Zhao et al., 2016a). Unlike BTS and the E3 ligase degrading FIT, *MdBT* expression and protein accumulation is induced when Fe availability is not limiting, leading to MdbHLH104 degradation (Zhao et al., 2016a). In addition, MdbHLH104 sumoylation, by the SUMO E3 ligase MdSIZ1, promotes MdbHLH104 stability, especially when Fe availability is scarce (Zhou et al., 2018). These findings suggest that the degradation of the bHLHs involved in the plant response to Fe availability may require two types of ubiquitin E3 ligases and that sumoylation may also have an important role in this process.

**FIGURE 2:**
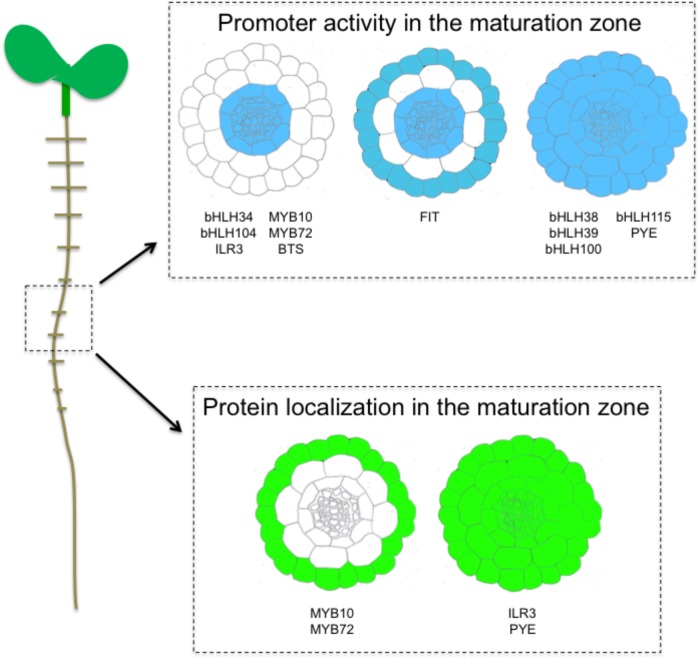
Schematic representation, based on previous studies, of expression (promoter activity) and localization (GFP fusion) pattern of transcription factors involved in the response to iron deficiency, in the maturation zone of the *A. thaliana* roots.

### bHLH Promoter Activity and Protein Localization

Iron uptake and translocation to the whole plant body requires coordinating the expression of several structural genes within and between the different cell types of roots that is achieved by the involvement of several TFs, in particular bHLHs (Figure 2).

Clade IVc bHLHs analysis of promoter activity revealed a specific expression in the stele (pericycle) of the root in the maturation zone (Li et al., 2016; Liang et al., 2017). Root tip expression was also detected except for *bHLH115*, whereas *ILR3* was the sole bHLH from this clade to be expressed in the elongation zone of root tips and early lateral roots. These differences suggest that non-redundant biological functions may exist between the members of this clade (Liang et al., 2017). For instance, ILR3 was shown to participate in the plant response to biotic and abiotic stresses (Samira et al., 2018). Perivascular expression has also been observed in the aerial part of the plants (i.e., hypocotyl and leaves) (Rampey et al., 2006; Zhang et al., 2015; Li et al., 2016; Liang et al., 2017). Promoter activity of *PYE* is similar in roots to that of *bHLH115*. In these cells PYE represses the expression of *NAS4* and *FRO3*, modulating Fe translocation to the above ground part of the plant (Long et al., 2010). Clade Ib bHLHs (except *bHLH101* whose promoter is not active in roots) were expressed throughout the root including the stele cells, which was in contrast with the expression pattern of their regulators, the clade IVc bHLH TFs (Wang et al., 2007). Clade Ib bHLHs promoter activity was essentially detected in the epidermis in the maturation zone, in the epidermis and inside the roots in the upper part zone (but not in the stele), and at the lateral root emergence site. However, a comparable pattern of expression with clade IVc bHLHs was observed in leaves. The apparent discrepancy observed in roots between the expression of clade Ib bHLHs and their transcriptional regulators suggests that clade IVc bHLH TFs may act in various cell types and thus have the ability to move from one cell type to another. In a recent study, it was shown that ILR3 protein is present in all root cell type when Fe availability is scarce (Samira et al., 2018). If this observation supports the hypothesis that ILR3 may move from the stele to other cell layers, it cannot be excluded that the promoter of *ILR3* is not sufficient for proper *ILR3* expression, and that sequences present in the coding region (e.g., introns) might be required. Interestingly, the pattern of PYE accumulation in root cells is similar to that of ILR3 (Long et al., 2010). The fact that ILR3 and PYE are present in the same cell layers and interact *in vivo* suggest that PYE function in the plant response to Fe deficiency might be tightly connected to ILR3 activity (Long et al., 2010; Selote et al., 2015). What would be the role of such heterodimers is still a matter of debate. Nevertheless, if the inter cellular trafficking of these two TFs was proven to be true, decrypting the underlying mechanisms would be the next challenge.

Fe-deficiency induced TF expression is mostly restricted to root tissues. At the rhizodermis, *FIT* expression overlaps those of its bHLH interacting partners from clade Ib (Colangelo and Guerinot, 2004; Jakoby et al., 2004). This is in agreement with the heterodimerization of FIT with clade Ib bHLHs to regulate the expression of Fe mobilization genes such as *IRT1* and *FRO2*. Two other TFs whose expression is partly regulated by FIT in response to Fe deficiency, namely MYB10 and MYB72, are mainly localized at the rhizodermis where they control the expression of *BGLU42* (Zamioudis et al., 2014). In the maturation zone, *FIT* is expressed in the epidermis and throughout the stele (Jakoby et al., 2004). Clade IVa bHLHs promoter activity is specific to the stele, a tissues where the repressive role of the encoded proteins on FIT stability is counteracted by FIT heterodimerization with clade Ib bHLHs (Cui et al., 2018). In the aerial part of the plant, clade IVa expression follows the vasculature as it is the case for ILR3 or clade Ib bHLHs.

## Conclusion

To date several TFs involved in the control of Fe homeostasis have been characterized and several molecular connections have been identified. However we still do not know how the expression of the most upstream TFs of this network is regulated (i.e., FIT, clade IVc bHLH), indicating that we still do not have the full set of TFs involved in this network. Whether or not the Fe deficiency and Fe excess responses are controlled by an integrated pathway involving common players is still an open question that remains to be addressed. Last, deciphering the precise localization, at the protein level, of all the TFs involved in the control of Fe homeostasis is an important task that should be achieved if one aims at fully decrypting the functioning of this intricate molecular network.

## Author Contributions

FeG, KR, FrG, EI, and CD wrote the manuscript. FeG, KR, and CD made the figures.

## Conflict of Interest Statement

The authors declare that the research was conducted in the absence of any commercial or financial relationships that could be construed as a potential conflict of interest.
